# Covid-19 pandemic? Mental health implications among nurses and Proposed interventions

**DOI:** 10.3934/publichealth.2024014

**Published:** 2024-03-11

**Authors:** Vasiliki Georgousopoulou, Panagiota Pervanidou, Pantelis Perdikaris, Efrosyni Vlachioti, Vaia Zagana, Georgios Kourtis, Ioanna Pavlopoulou, Vasiliki Matziou

**Affiliations:** 1 Department of Research, Quality Control and Continuing Education, University General Hospital of Alexandroupolis; 2 First Department of Pediatrics, Medical School, National and Kapodistrian University of Athens, “Agia Sophia” Children's Hospital; 3 Department of Nursing School of Health Sciences University of Peloponnese; 4 Department of Nursing, ‘Aghia Sophia’ Children's Hospital, Athens, Greece; 5 Department of Nursing, “Sotiria” General Hospital, Athens, Greece; 6 Department of Nursing, National and Kapodistrian University of Athens

**Keywords:** healthcare workers, stress response, psychopathology, psychological problems, COVID-19, mental health, nurses

## Abstract

**Background:**

With its abrupt and huge health and socio-economic consequences, the coronavirus disease (COVID-19) pandemic has led to a uniquely demanding, intensely stressful, and even traumatic period. Healthcare workers (HCW), especially nurses, were exposed to mental health challenges during those challenging times.

**Objectives:**

Review the current literature on mental health problems among nurses caring for COVID-19 patients.

**Methods:**

This is a narrative review and critical evaluation of relevant publications.

**Results:**

Nurses experienced higher levels of stress, burnout, anxiety, depression, frustration, stigma, and depersonalization compared to other HCW. Factors that increased this symptomatology included concerns about infection or infection of family members, inadequate staff protective equipment, extended working hours, insufficient information, a reduced sense of security, and post-traumatic stress disorder. The factors that improved the psychopathology included a general positive attitude, job satisfaction, adequate information and education, harmonious group relationships, post-traumatic development, emotional intelligence, psychological counseling, mindfulness-based stress reduction, stable leadership, guidance, and moral and practical administrative support.

**Conclusions:**

Recent studies clearly show that nurses, especially women, are the most vulnerable subgroup among HCW and are particularly prone to mental health impacts during the COVID-19 pandemic. The documented mental health vulnerability of frontline nursing staff during the COVID-19 pandemic requires preventive nursing management actions to increase resilience and to develop relevant defense mechanisms.

## 1. Introduction

Exposure to adverse environmental and/or intrinsic conditions (actual or perceived) triggers both physical and mental responses, collectively known as the stress response. The goal of the stress response is to neutralize stress and restore homeostasis [1]. The type, intensity, and duration of stressors, as well as the person's biological and psychological background, previous relevant experiences, and available support, influence the outcome of this “battle” [2].

Shortly after the coronavirus disease (COVID-19) was officially declared a “public health emergency” (and later a pandemic), and while emerging shortages in healthcare facilities led to global lockdowns, it became clear that these unprecedented circumstances could increase the incidence of stress-related psychological disorders [3]. In particular, although anyone can experience mental or emotional problems related to COVID-19, certain groups, such as healthcare workers (HCW), especially nurses, appear to be more vulnerable to developing psychopathology within or after the pandemic [4]–[6]. In addition, we must not forget that the work-related burnout of nurses has a direct impact on the quality of patient care [7].

Therefore, reviewing literature references on emerging psychopathologies in nurses caring for COVID-19 patients was considered important for both personal and public health. Such a review can help to not only identify specific harms and problems, but also to identify the interventions needed to prevent and/or manage such potential consequences.

## 2. Methods

Articles were searched using PubMed with the search terms “{(COVID-19) AND [(nurses) OR (healthcare workers)] AND [(mental health) OR (psychological problems) OR (psychopathology)]}”. Articles in English with full-text availability were filtered, resulting in 3,913 matches, that were further evaluated using relevant subjective criteria (due to excessive article numbers and extensive variability) such as relevance, representativeness, sample size, methodology, and reliability. First, abstracts were assessed for relevance with our main points of interest (i.e., mental health implications among nurses vs other types of HCW as well as the general public). Furthermore, publications were evaluated with respect to the number of participants (including large scale studies), the validity of the assessment tools used, timing (so that publications are were of the main phases of the pandemic including, but not limited to, the deadliest first wave), and origin of research (in an attempt to include a wide range of countries). Finally, publications were selected in an attempt to cover specific subjects of special interest such as an immediate impact, long-term effects, the role of gender, the role of employment differences (first responder, ICU etc.), the type of position, the role of the availability of protective equipment, and protective factors.

## 3. Discussion and results

### 3.1. Impact of the COVID-19 pandemic on nurses' mental health

Globally, psychological distress and burnout among nurses pose a significant threat to healthcare systems, especially in the context of stressors related to the COVID-19 pandemic. Recognizing this risk, several studies have examined the impact of the COVID-19 pandemic on nurses' mental health. As expected, Chinese scientists provided the majority of initial literature data on mental health impacts among nurses in the COVID-19 era. Lai J et al. (2020) investigated the factors affecting the mental health of 1257 HCW from 34 Chinese hospitals during the COVID-19 pandemic. Of the participants, 60.8% were nurses, 39.2% were medical doctors, and 41.5% were first-line HCW. The reported symptoms included depression (50.4%), anxiety (44.6%), discomfort (71.5%), and insomnia (34%). Nurses, women, and frontline HCW working in Wuhan, China, reported more severe mental health symptoms compared to other participants in the entire HCW group. Nurses scored higher than medical doctors, and women reported more severe depression, anxiety, and discomfort than men [8]. Nie A et al. (2020) examined the psychological impact of COVID-19 on 263 frontline nurses in China and found that 25% of them experienced psychological distress, which was higher than the general population in China over the same period [9].

Shahrour G et al. (2020) investigated the prevalence of acute stress disorder (ASD) and predictors of psychological distress among 448 Jordanian nurses during the COVID-19 pandemic. Overall, 64% of nurses experienced ASD and were at risk for post-traumatic stress disorder (PTSD), while 41% of them reported significant psychological distress, with younger nurses being more susceptible. Nurses with higher ASD scores experienced more psychological distress, while coping skills and self-efficacy were protective factors [10].

A number of studies compared frontline nurses (who were exposed to COVID-19) and nurses working in regular departments (who were not exposed to COVID-19). For example, Sarboozi Hoseinabadi T et al. (2020) found that among 245 nurses working in a hospital in Iran, work-related stress and burnout in the exposed group were significantly higher than those in the unexposed group [11]. In another study, Di Tella M et al. (2020) examined the psychological impact of COVID-19 among 145 Italian HCW (72 medical doctors and 73 nurses). Comparing the responses of HCW working in COVID-19 wards with those working in other units, it was shown that those working in the COVID-19 wards reported higher levels of depression and PTSD. Therefore, it is important to identify specific factors that predispose HCW in the care of patients with COVID-19 to the development of psychopathology in order to develop targeted intervention strategies [7]. In contrast, Liu Y et al. (2020) found that the overall incidence of mild to moderate distress among second-and first-line nurses was 31% and 25%, respectively. In addition, the incidence of severe discomfort did not differ between the two groups. The interpretation of this paradoxical finding is not completely clear, but the findings are interesting and useful, since the authors suggest that frontline nurses might be less distressed due to living in separate government-appointed accommodations rather than their own home (therefore, protecting their families), had more protective equipment, and more available information compared to second-line nurses. Thus, protective measures have more of an impact than actual exposure to COVID-19 [12].

Following these initial reports, the Health Sciences Foundation (Spain) reported an increase in mental health challenges among HCW during the COVID-19 pandemic, influenced by factors such as being a woman, working as a nurse, proximity to COVID-19 patients, working in rural settings, and psychiatric history [13]. Similarly, Harris ML et al. (2023) revealed that the COVID-19 pandemic had adverse effects on nurses' mental health, including feelings of isolation, loss, strong emotions, and a sense of worthlessness. In addition, they reported physical health problems, including exhaustion and skin problems, due to the prolonged use of personal protective equipment (PPE) [14]. Another study by Vázquez Sánchez MÁ et al. (2023) analyzed 25 Spanish nurse interviews to investigate the professional grief experienced by nurses during the COVID-19 pandemic and its impact on nurses' psyche, and consequently on their professional and personal lives. They found that a significant number of nurses experienced professional grief, often accompanied by a range of related symptoms. Additionally, this study highlighted the importance of coping with grief, providing appropriate training and proactive support for nurses to cope with the effects of patient deaths, and changes in their work environment [15]. In this regard, Stubin CA et al. reported that resilience training could improve nurses' stress levels and attention, thus leading to improved patient outcomes. In addition, it can contribute to the development of innovative strategies that promote career longevity and increase overall job satisfaction [16].

Moral distress among nurses is another issue that needs to be addressed. Alimoradi Z et al. (2023) found that nurses who worked in developing countries had higher levels of moral distress compared to those in developed countries, in which the working conditions did not significantly affect the severity of distress. This study highlighted the need for healthcare institutions and future research to create supportive environments for nurses to mitigate moral distress [17].

Beck JG et al. (2023) examined the impact of stressors such as exposure to COVID-19 patients, patient death testimony, living separately from their family for safety, institutional betrayal (i.e., the perception that an institution failed to protect a member who depends on and trusts it), and the perception of institutional failure to protect members in the mental health of 391 nurses. The findings showed that institutional betrayal independently led to symptoms PTSD, generalized anxiety disorder (GAD), and major depressive disorder (MDD) among caregivers, but did not significantly change the relationship between stressors and mental health. Higher exposure to COVID-19 patients was associated with more severe symptoms of PTSD, GAD and MDD in nurses [18]. Boone LD et al. (2023) conducted a comprehensive review of literature published between March 2020 and February 2021, focusing on factors that affect nurses' well-being and safety during the pandemic, as well as interventions to promote their mental health. They found that nurses experienced loss of life (i.e., patients, unexpected personal, and professional losses), hope, and professional identities during the pandemic. In addition, many nurses reported a lack of visible and supportive leadership in healthcare organizations that contributed to increases in anxiety, stress, depression, and moral distress. These findings highlight the importance of implementing comprehensive support mechanisms and effective leadership strategies to address mental health challenges faced by nurses during crises [19]. Similarly, a meta-analysis of 401 studies, which involved 458,754 participants in 58 countries [20], revealed the following aggregate prevalence rates among hospital HCW: depression at 28.5%, anxiety at 28.7%, post-traumatic stress disorder (PTSD) at 25.5%, alcohol and substance use disorder at 25.3%, and insomnia at 24.4%. These prevalence rates differed between different categories of HCW, including physicians, nurses, allied health professionals, support staff, and healthcare students. Specifically, there were significantly higher odds of potential mental health disorders among women, those working in high-risk units, and those directly involved in patient care. However, the majority of the included studies were based on self-reporting measures, which mainly reflected possible mental health disorders rather than confirmed diagnoses [20].

In addition, sleep disturbances were identified in a large HCW cohort (10,467 HCW longitudinally and 3313 longitudinal) [21]. They found that factors such as gender, previous diagnoses of mental illness, and COVID-19 frontline work were associated with higher sleep disorder scores. This study emphasized the importance of addressing HCW mental health needs, especially those with prior diagnoses of mental illness, to mitigate insomnia during the COVID-19 pandemic [21]. Moreover, other studies have shown the importance of interventions, including organizational support, psychosocial support, and enhancing psychological flexibility, which can mitigate the adverse effects of burnout [22]–[25]. In addition, educational attainment, job satisfaction, intrinsic motivation, and loyalty (i.e., “we are family” theme among HCW and value of supportive collegial relationships as well as supportive working environments) play an important role in coping with stress related to COVID-19 [22]–[25].

He C et al. (2023) investigated the prevalence and risk factors of somatization, depression, and anxiety among frontline nurses during the early phase of the COVID-19 pandemic. They found significant psychological distress, with hospitalization levels (provincial versus county-level hospitals) and the length of employment as independent risk factors for stress disorders among these nurses, with provincial level nurses reporting decreased somatization, depression, and anxiety [26]. A cross-sectional study by Che H et al. (2023) investigated the relationship between the long working hours and the mental health of 2811 Chinese nurses who worked in a tertiary hospital in China during the COVID-19 pandemic. Key findings showed that extended working hours were associated with an increased risk of mental disorders among nurses during the pandemic, especially among those who work more than 60 hours per week. A significant proportion of respondents reported symptoms of depression (7.80%) and anxiety (6.70%) [27].

Sagherian K et al. (2023) evaluated the impact of the COVID-19 pandemic on 587 hospital nurses and nursing assistants in the United States, focusing on insomnia, fatigue, intershift recovery, and psychological well-being. The findings revealed that the nursing staff experienced sleep disturbances, significant chronic fatigue, increased emotional exhaustion and depersonalization, moderate psychological distress, and high post-traumatic stress. Factors such as long working hours and short breaks were associated with these negative outcomes [28]. Studies by Che et al. (2023) and Sagherian K et al. (2023) highlighted the importance of managing working hours and providing support to protect HCW mental well-being during health crises [27],[28]. Mao X et al. (2023) examined the mental health of female nurses in China during the normalization of COVID-19 prevention efforts. Among the 740 participants, 7.9% experienced anxiety and 17.8% experienced depression. Insomnia was linked to anxiety and depression, and PTSD symptoms were associated with these mental health problems. Marriage was found to be a protective factor against depression. The study highlighted the importance of addressing the mental health challenges faced by nurses, particularly those who were single, and implementing strategies to improve their sleep quality and manage stress associated with traumatic events [29].

Credland N et al. (2023) interviewed 54 critical care nurses from 38 hospitals in the UK and Ireland to explore the mental health challenges they faced during the COVID-19 pandemic. They found that nurses felt powerless in managing the crisis. Witnessing pain and critical care demands negatively impacted their mental health. Unexpectedly, they found themselves in leadership roles, thus adding to their stress. Moreover, despite public and political praise, the lack of practical support, including equipment, leadership, emotional support, and fair compensation, had a detrimental long-term impact [30].

Spruijt I et al. (2023) conducted a study that included semi-structured interviews with COVID-19 HCW from five different countries. The findings showed that COVID-19 HCW in these countries faced stigma, primarily due to fears of infection and the perception that they may carry the disease. Stigma manifested itself differently in each country and included reprimand, discrimination, avoidance, and isolation. These experiences led to feelings of depression, loneliness, and a desire to quit their jobs, thus highlighting the significant challenges HCW faced during the pandemic [31].

Vianna ECDC et al. (2023) conducted a qualitative study which focused on nurses in Brazil's emergency services during the COVID-19 pandemic. Through virtual face-to-face interviews, they investigated the perceived health effects. The study revealed various physical, mental, and psychosocial alterations among professional nurses, including eating disorders, fatigue, burnout, anxiety, and social isolation [32].

Ergin E et al. (2023) conducted a study to assess nurses' stress levels during the peak of the second wave of the COVID-19 pandemic in Turkey and explored the use of coping methods, including complementary and alternative medicine (CAM). The study found that nurses typically used methods such as prayer, hot/cold showers, and herbal teas to cope with stress [33].

The study by Baraka AAE et al. (2023) aimed to identify predictors of stress, anxiety, and depression among critical care nurses in response to the COVID-19 pandemic in Alexandria, Egypt. The study found important predictors of each psychological impact. The stress was predicted by the number of infected colleagues and the availability of hospital resources. Stress was associated with factors such as age, gender, income satisfaction, years of experience, time spent caring for COVID-19 patients, continuing education, the number of infected colleagues, and the availability of hospital resources. Depression was predicted by gender, history of physical problems, educational attainment, availability of hospital resources, history of psychological problems, and number of infected colleagues. These findings highlight the importance of identifying these prognostic factors for developing mental health promotion strategies that can provide critical support to nurses during the ongoing pandemic [34].

Almhdawi KA et al. (2023) conducted a study to assess the health-related quality of life (HRQoL) of nurses during the COVID-19 pandemic in Jordan and to identify the key health and work factors affecting it. The study found that nurses who reported better sleep quality self-assessments tended to have higher HRQoL levels. In contrast, increased levels of depression, musculoskeletal pain, and financial burden on their families were associated with lower HRQoL levels among Jordanian nurses [35].

Yarifard K et al. (2023) investigated work-family conflict (WFC) and burnout among nurses in northwestern Iran that involved 256 nurses who worked in COVID-19 referral hospitals. Key findings included a significant level of conflict, with the highest scores in the time dimension of WFC and burnout, mostly characterized by a lack of personal fulfillment. All aspects of WFC, emotional exhaustion, and depersonalization correlate positively with burnout. Factors such as ward, hospital, and employment status affected the WFC. Interestingly, taking a crisis management course was associated with more severe depersonalization and a higher incidence of lack of personal fulfillment. The frequency and severity of emotional exhaustion were related to employment status and work experience. Therefore, improving working conditions and providing organisational support can address the higher-than-average levels of conflict and burnout among nurses [36].

Kealeboga KM et al. (2023) conducted a qualitative case study in Botswana to investigate the psychological distress experienced by nurses working in COVID-19 special care centers during the pandemic. Through semi-structured phone interviews with nurses from select COVID-19 centers in Gaborone, they found that nurses experienced feelings of fear, anxiety, hopelessness, helplessness, loneliness, and physical discomfort. This study highlights the importance of recognizing and addressing the mental and emotional well-being of nurses working in high-stress environments [37].

A cross-sectional study by Khatatbeh H et al. (2023) investigated burnout levels, the quality of life (QOL), and perceptions of patient-related adverse events among pediatric nurses during the COVID-19 pandemic. The findings showed high levels of burnout among pediatric nurses, a low quality of life, and a high incidence of nosocomial infections. The age of nurses and the inpatient capacity of the hospital or hospital unit in which they worked were significantly associated with burnout and the quality of life [38]. Therefore, specific measures should be taken to address their workload and financial concerns, as well as to strengthen the resilience of nurses. Hwang S et al. (2023) examined the impact of resilience in Intensive Care Unit (ICU) nurses during the COVID-19 pandemic, thus highlighting its role in alleviating depression and burnout. Their findings highlight the importance of resilience in mitigating the adverse effects of increased stressors and workload in these healthcare professionals [39].

The studies presented above, as well as those included in the systematic review conducted by García Vivar C et al. (2023), show that a significant proportion of nurses, particularly those working in hospital and frontline settings, suffer moderate to severe symptoms of anxiety, depression,PTSD, and insomnia. Therefore, it is important to assess and address the mental well-being of nurses in various healthcare settings and to develop supportive measures [40].

### 3.2. Long-term effects of COVID-19 on nurses' mental health

A longitudinal study conducted in Portugal showed that depression, anxiety, stress, and poor sleep quality showed a downward trend over time in nurses caring for COVID-19 patients (Sampaio F et al. 2023). Gender, age, nurse specialty, number and quality of PPE, and fear of infecting or infecting others were all possible predictors of changes in the depression and anxiety scores over time, as well as the fear of displacement from residence. Males had lower stress levels than females, as did elderly and skilled nurses. Moreover, the greater the fear of infecting or infecting others, the more stress symptoms were present. In terms of sleep quality, the number of nurses with a poor sleep quality significantly decreased over time [41].

Chen R et al. (2021) conducted a large-scale survey to investigate trauma, burnout, and post-traumatic development in 12,596 nurses with a high percentage (76.5%) of university or tertiary education and an average nursing experience of 10.4 years. At the conclusion of the survey, 13.3% of the participants reported trauma, thereby describing moderate degrees of emotional exhaustion, while 39.3% experienced post-traumatic development, which signaled the positive psychological state they experienced after struggling with extremely adverse life conditions. Therefore, not all people who experienced trauma experienced long-term despair. Emotional exhaustion and mental health problems were higher in women and hospitals designated for COVID-19 [42].

The study by Sorokin MY et al. (2020) evaluated the severity of psychological distress and stigma in different classes of HCW caring for COVID-19 patients. A total of 1800 Russian-speaking individuals were evaluated using data collected over the following two time periods: March 30 to April 5 and May 4 to May 10, 2020. Overall, the levels of psychological stress decreased in the second phase, while the levels of stigma increased [43]. Medical doctors experienced more stress than nurses and paramedics and were less likely to be stigmatized. Stigma reactions were more prevalent among nurses and paramedics and were not directly related to the risk of infection. Additionally, the sense of stigma was emphasized in a study by Azizpour I et al. (2021), which included 312 nurses in Iran. A positive relationship between the average stress score and stigma was revealed [44].

Costa A et al. (2023) conducted mental health assessments of HCW in Portugal during the COVID-19 pandemic. These evaluations included cross-sectional and longitudinal assessments in 2020 and 2021, respectively. Although the rate of moderate to severe symptoms decreased over time, the findings showed that a significant proportion of HCW continued to experience discomfort. The risk factors included participation in women, work in COVID-19 frontline positions, and work-life balance challenges. The protective factors included a high resilience, a strong social support, maintaining hobbies, and a healthy lifestyle [45].

The study by Padmanathan P et al. (2023) investigated suicidal thoughts and behavior (STB) among National Health Service (NHS) HCW in England during the COVID-19 pandemic. The survey found that about 10.8% of HCW reported suicidal thoughts in the previous two months, and 2.1% reported suicide attempts. In addition, 11.3% of HCW who initially reported suicidal thoughts experienced them six months later. Factors associated with increased suicidal ideation included exposure to morally harmful events (using the Moral Injury Events Scale which helps report a person's feelings during unspecified events that one thinks are morally wrong), lack of confidence in increasing safety concerns, feeling unsupported by managers, and the provision of reduced standards of care [46].

Changes in compassion fatigue, burnout, compassion satisfaction, and fear of COVID-19 before and after the COVID-19 vaccination campaign was conducted among Spanish nurses. Data was collected from 439 registered nurses in December 2020 and 410 in December 2021 using a repeatable cross-sectional design spanning 12 months. The fear of COVID-19 remained persistent among nurses, with consistent and high levels of burnout. In particular, compassion fatigue and satisfaction decreased. Positive associations were found between a fear of COVID-19 and burnout, as well as between a fear of COVID-19 and compassion fatigue. Negative associations were found between fear and compassion satisfaction. The study highlighted the need for programs to reduce COVID-19 fear, burnout, and compassion fatigue among nurses to enhance their mental health, prevent psychological distress, and maintain the quality of nursing care [47].

The exploratory longitudinal study “Well-being of healthcare workers [Benessere Operatori Project]” assessed the mental health of 325 Italian HCW in various roles during the COVID-19 pandemic at three different points over a 14-month period. The findings showed that participants generally reported an increase in the subclinical levels of psychiatric symptoms, such as depression, anger, and emotional exhaustion, that remained relatively stable over time, with a few exceptions. Despite the subclinical nature of the symptoms, this study highlights the importance of addressing HCW distress, as it can potentially affect the quality of care, patient satisfaction, and medical error rates [48].

Heesakkers H et al. (2023) investigated the impact of the second wave of the COVID-19 pandemic on the mental well-being of ICU nurses in the Netherlands. In an online survey, 38.2% of nurses reported experiencing one or more mental health symptoms, and 49.9% reported work-related fatigue. Comparing the two time points, the mental health symptoms remained high, while work-related fatigue significantly increased. Factors associated with fewer mental health symptoms included sponsored vacations, confidence about the future, and an improved perceived work-life balance [49].

The studies described above highlight the ongoing long-term challenges faced by nurses during the COVID-19 pandemic, which may adversely affect their mental health and well-being, as well as patient care and the health system stability. As such, HCW face ongoing mental health challenges, thus highlighting the critical need to receive support and build resilience in a pandemic.

### 3.3. The role of gender and employment differences

The systematic review and meta-analysis by Pappa S et al. (2020), which covered up to April 2020, included 13 studies with a total of 33,062 participating HCW. The pooled prevalence of anxiety was 23.2% (20.92% in men and 29.06% in women), depression was 22.8% (20.34% in men and 26.87% in women), and insomnia was 34.32%. The prevalence of depression was 30.3% for nurses and 25.37% for medical doctors; moreover, anxiety was 25.8% for nurses and 21.73% for medical doctors. Therefore, the analysis revealed significant differences between genders and occupations, which are factors to consider when designing interventions for improvements [50].

Ruiz Fernández MD et al. (2020) assessed compassion fatigue, burnout, compassion satisfaction, and perceived stress among 506 medical doctors and nurses working in healthcare centers during the highest incidence period of COVID-19 cases in Spain. Medical doctors reported higher compassion fatigue and burnout scores than nurses, while nurses had higher compassion satisfaction scores (similar to those reported before the crisis). The perceived anxiety did not differ between medical doctors and nurses. Remarkably, despite having perceived stress levels comparable to those among medical doctors and working in equally challenging conditions, nurses had significantly higher compassion satisfaction scores than nurses reported in studies conducted in the same Spanish setting before the COVID-19 pandemic [51]. Similarly, Lai J et al. (2020) reported that nurses, women, and HCW working in Wuhan, China, suffered from severe mental health symptoms more than other participants, including medical doctors, and women reported more severe depression, anxiety, and discomfort than men [8]. Badahdah A et al. (2021) also reported a high prevalence of stress, anxiety, and poor psychological well-being, especially among HCW women in Oman (315 nurses and 194 medical doctors) [52].

Barello S et al. (2020) conducted a study involving 67 medical doctors and 271 nurses working in Italian hospitals. They found that the level of emotional exhaustion was significantly higher than in the normative sample, while levels of depersonalization were lower and those of personal satisfaction were higher. The analysis of the factors that influenced the various psychopathological manifestations showed once again that gender played an important role, with women experiencing greater levels of burnout compared to men. Job satisfaction was reported by a high percentage of participants and was found to be beneficial for mental health [53]. Despite differences in study design and, in particular, outcome measures, it is clear that nurses, especially female nurses, are the most vulnerable group to the COVID-19 pandemic in terms of stress and mental health outcomes, not only compared to the general population, but also among HCW and across nations [54]–[62].

The study by Brady C et al. (2023) during the third wave of the COVID-19 pandemic examined the mental health of 377 hospital workers in Dublin, Ireland, including medical doctors, nurses, and radiologists. The results showed that 45.1% of participants experienced moderate to severe symptoms of post-traumatic stress disorder. As indicated by a low WHO-5 well-being index score, low mood was reported by 52% of participants and 13% reported one-week suicidal ideation. The work capacity was insufficient for 39% of staff members. While differences were observed between occupational groups, medical doctors generally reported fewer symptoms and fewer moral injuries (i.e., the distress experienced when an individual witness or engages in acts that contradict their moral and ethical beliefs [63].

Furthermore, the impact of the COVID-19 pandemic on the mental health and sleep habits of Italian HCW, especially nurses and medical doctors, were evaluated by Alfonsi V et al. (2023). The findings revealed that both groups were negatively affected by the pandemic, though nurses experienced a greater distress, with a significant worsening of various psychological and sleep-related symptoms over time [64].

A quantitative study by Tamrakar P et al. (2023) conducted in Nepal compared the prevalence of anxiety and depression among nurses working in ICUs with COVID-19 (COVID ICU) and non-COVID ICU patients. The study found that a high percentage of nurses in both types of ICUs developed a psychiatric caseness (i.e., scored highly enough on measures of depression and anxiety so it could be classed as a clinical case), thus indicating potential mental health challenges. While some factors differed, such as confidence in caring for COVID-19 patients and intentions to interrupt their current work, the prevalence of anxiety and depression did not significantly differ between the two groups [65]. The study by Hegazy AA et al. (2023) focused on the psychological impact of the COVID-19 pandemic on the medical staff in neonatal intensive care units (NICUs) in pediatric hospitals affiliated with Cairo University in Egypt. The majority of participants reported fairly safe levels of anxiety (i.e., according to the work stress questionnaire score, a questionnaire developed by the Indian Council of Medical Research). However, female participants and medical doctors had higher average scores for anxiety symptoms compared to other groups [66].

The psychological impact of the COVID-19 pandemic on HCW in Bangladesh was investigated by He C et al. (2023), where the authors particularly focused on the prevalence of insomnia and work-related stressors. The research found a significant prevalence of clinically significant insomnia, in which medical doctors and nurses experienced higher rates than other HCW. Factors such as sick leave, entitlement to risk benefits, and a previous COVID-19 diagnosis affected the likelihood of insomnia. The study highlighted the importance of addressing sleep-related issues and risk factors for HCW [26].

In the study by Martin SD et al. (2023), nursing managers in the United States were asked about their well-being during the pandemic. The findings revealed high levels of stress, with many nursing managers experiencing work-related stress spilling over into their personal lives, thus contributing to burnout and emotional problems. The study highlighted the need for interventions to improve the well-being of nursing managers, including administrative support and policy changes [67].

In a study by Canal Rivero M et al. (2023), the impact of the COVID-19 pandemic on HCW mental health was examined, focusing on gender differences. They examined 1432 HCW in May 2020 and 251 of them again in November 2020. The findings showed that HCW experienced worsening physical symptoms over time. Gender differences were significant across all dimensions of the questionnaire and the overall score. There was a significant interaction between time and gender, particularly in physical and anxious dimensions. Stress related to the spread of COVID-19 and work-induced crashes were primary predictors of psychological distress. Different predictors were associated with distinct dimensions of psychological distress, thus revealing the complexity of addressing mental health issues in high-risk populations during major crises. In conclusion, while physical symptoms increased during the pandemic, initial gender inequalities in psychological distress decreased as the pandemic continued [68].

Kells M et al. (2023) conducted a cross-sectional study to assess the impact of the COVID-19 pandemic on undergraduate nursing students in the US. Their research aimed to explore the student's emotional state, life satisfaction, stress levels, media consumption, and perceptions of pursuing nursing careers during the pandemic. The study found that nursing students reported significant stress, with many feeling overwhelmed and anxious about the future. A remarkable percentage experienced moderate to severe anxiety and depression. In addition, the majority reported that COVID-19 affected their interest in nursing. This study shed light on the multifaceted impact of the pandemic on the well-being and career choices of nursing students [69].

### 3.4. The role of personal protective equipment (PPE)

The availability (or lack off) and the quality of PPE has a great impact on the effect of COVID-19 on mental health. Bruyneel A et al. (2021) studied 1,135 nurses from Belgium and reported a 68% overall prevalence of burnout and 29% of depersonalization, with a higher risk associated with the work overload and the lack of PPE [58]. Similarly, in a study by Sampaio F et al. (2021) in Portugal, nurses who were satisfied with the quantity and quality of PPE experienced significantly lower levels of depression, anxiety, and stress compared to nurses who considered the PPE to be inadequate. Nurses who worked longer hours had higher levels of depression, anxiety, and stress. Additionally, a significant positive correlation was observed between the fear of infection or infection of family members and levels of depression, anxiety, and stress [70].

In a study by Hendy A et al. (2020), 374 nurses from five COVID-19 hospitals in Egypt were included, with 52.1% of participants reporting moderate levels of overall stress, 26.2% as severe, 13.4% as mild, and only 8.3% as normal stress levels. The negative predictors of stress were COVID-19 education, PPE availability, and the attention from hospital management. The positive predictors of stress were marital status, having children, people showing COVID-19 as a stigma, the fear of infection, a low nurse-to-patient ratio, and the fear of transmitting the infection to the family [71].

The study by Saifullah et al. (2023) investigated the impact of the COVID-19 pandemic on HCW in public hospitals in Sindh province, Pakistan. Through qualitative interviews with 320 HCW from 10 hospitals, a number of challenges were revealed, such as an inadequate hospital infrastructure, a lack of protective equipment, and a lack of isolation facilities. These difficulties caused physical and psychological stress, thus resulting in fatigue, sleep disturbances, mental stress, and an increased fear of infection among HCW [72].

### 3.5. Protective factors and strategies

The documented mental health vulnerability of frontline nursing staff during the COVID-19 pandemic highlights the need for proactive nursing management strategies aimed at strengthening resilience and building support mechanisms. Understanding the factors that affect nurses is essential to developing effective coping mechanisms. A study by Soto Rubio A et al. (2020) investigated the effect of psychosocial risk and emotional intelligence on nurses' health, well-being, burnout level, and job satisfaction during the peak of the COVID-19 pandemic in Spain. Emotional intelligence is defined as the ability to recognize, understand, and manage one's emotions as well as those of others, thus distinguishing between them and using this knowledge to guide one's thoughts and actions. It is fundamental for communication, empathy, sensitivity, creativity, self-awareness, self-control, and self-confidence. Nurses who are armed with a greater emotional intelligence have a greater ability to listen to patients and care for them. In this study, 125 nurses were evaluated. It was found that the psychosocial risk and emotional intelligence predicted 50% of the variation in burnout, 41% of the variation in job satisfaction, and 32% of the variation in psychosomatic health problems. The data show a protective effect of emotional intelligence against the adverse effects of psychosocial risk, such as burnout and occupational stress, and show that it exerts favorable effects on job satisfaction. These findings can contribute to the development of appropriate intervention programs [73].

Stressors and adaptation strategies among 127 nurses in Ecuador were assessed by Franco & Leví (2020). Stressors included the likelihood of transmitting the disease to relatives (in 90%), workplace-related factors, concern about infection from handling patients (94%), a lack of PPE (91%), and a lack of specific treatment and vaccines (91%). A positive attitude (reported by 100% of participants) was a factor that helped reduce stress. The same percentage was reported in terms of teamwork, knowing that COVID-19 cases were improving and knowing that no one was sick in their family or among friends (100%). Eighty-four percent of participants noted that avoiding long working hours was beneficial. The authors concluded that the impact of COVID-19 on the mental health of nurses could be severe and that social and family components favorably influence their coping capacities. Strikingly, nurses prioritized humanitarian feelings and their sense of professional duty, particularly among young people (59% of those aged 35). It has been reported that their professional identity was affected by stress, with nurses showing a declining professional identity as stress increased [74].

The study by Nowicki GJ et al. (2020) investigated the level of post-traumatic stress, perceived social support, views on the positive and negative consequences of the pandemic, a sense of security, and a sense of meaning in life (a sense considered essential for human existence) among 325 Polish nurses facing this epidemiological storm. A high level of PTSD was reported with very pronounced avoidance symptoms, alongside a reduced sense of security related to their personal safety and that of others. Interestingly, a well-prepared workplace helped to increase the sense of security. A sense of meaning in life characterized the group, although the tendency to seek it out was less apparent. The highest rates of support came from the “significant other” (i.e., a special person in one's life, other than family and friends according to the Multidimensional Scale of Perceived Social Support) [75]. Similarly, Shahrour G et al. found that nurses with higher acute stress disorder scores experienced more psychological distress, while those with coping skills and self-efficacy were more protected. Therefore, it is crucial to identify this situation and implement appropriate preventive measures as soon as possible [10]. In support of this, Liu Y et al. (2020) found that a higher perceived social support was the main protective factor for acute stress disorder, while a negative coping style was a risk factor [12].

It should be emphasized that the pre-pandemic prevalence of burnout syndrome among registered nurses in the U.S. was reported to range from 35 to 45%. In one study, nurses had twice the rate of depression compared to other HCW. In general, nurses are more likely to have higher stress levels, less sleep, and a greater likelihood of being overweight compared to the general population. In the era of the COVID-19 pandemic, burnout is a major threat to the stability of the frontline workforce [76]. In a review article by Janeway in September 2020, emphasis was placed on the role of psychiatry in treating burnout among nurses during the COVID-19 pandemic. Psychiatric consultations can provide support through liaison meetings, stress management programs, and sidewalk consultations, which are interventions that can help reduce the risk of burnout. However, in this regard, there is an urgent need to overcome the stigma of psychiatric consultations and make such interventions easily accessible. Stress reduction and mindfulness-based meditation are additional means to reduce stress [77].

It is important to note that in addition to physical and emotional stress, nurses and other HCW, especially emergency workers, also experience moral distress resulting from situations that put them in conflict with their professional values. In other words, they struggle not only with long and exhausting working hours, but also with complex ethical issues. Therefore, it is evident that nurses urgently need strong moral support to strengthen their resilience, especially those working on the frontline during the COVID-19 pandemic. In addition, high resilience, spirituality, and family functioning seem to represent good coping mechanisms for nurses against stress, anxiety, and depression during the pandemic [78],[79].

A study involving 500 nurses working in Saudi Arabia was conducted by Al Dossary R et al. (2020), in which 96.8% of participants reported excellent knowledge of COVID-19. Only 7.6% reported that their knowledge about prevention was limited and 60.4% had a high positive attitude towards caring for COVID-19 patients. Women nurses, married nurses, and those with a bachelor's degree had a greater awareness, better attitudes, and better clinical experience with prevention. It is worth noting that 65.4% of nurses received the COVID-19 health information from the Ministry of Health [80].

In the age of COVID-19, nurses need strong leadership, clear directions, and ongoing support from each other, their employers, the public, and nursing organizations [81],[82]. Reports that elaborate strategies and resources for nurse leaders [9],[83] state that it is vital to guide nurses with empathy and prudence in the complex circumstances of the COVID-19 pandemic and other similar circumstances. Moreover, it is vital that nurse leaders ensure that frontline nurses are properly prepared to provide safe and high-quality care to COVID-19 patients, as well as end-of-life care if necessary. In addition, individualized self-care programs must be implemented to reduce nurses' stress due to care in all these difficult cases. Finally, nurse leaders are advised to motivate the team by implementing the following three strategies: providing direction, creating meaning, and empathy. The HCW request to its leaders can be summed up as follows: “Listen to me, protect me, prepare me, support me, take care of me.” [84].

Lin CH et al. (2023) conducted a scoping review that focused on a cognitive assessment and stress-related coping strategies among registered emergency department nurses during the COVID-19 pandemic. The review of sixteen studies highlighted the overwhelming challenges faced by emergency department nurses, including depression, screening discomfort, and physical exhaustion. The need for comprehensive training, modified screening systems, safe workplaces, psychological support, and resilience promotion is stressed to help nurses working in such an environment, effectively address pandemic-related challenges, and prioritize their wellbeing [85].

Moreover, a comprehensive review that analyzed qualitative research on nurses' psychological experiences while caring for COVID-19 patients was conducted by Lim S et al. (2023). The study identified both negative and positive psychological experiences of nurses and stressed the importance of integrated support that included psychological, social, economic, and organizational aspects to enhance nurses' mental well-being and the quality of nursing care [86].

The mental health of 711 registered nurses during the COVID-19 pandemic was assessed and the roles of social support and psychological resilience as mediators between coping strategies and mental health were examined by Xu Y et al. (2023). The study found that many nurses were at a high risk of mental health problems. It highlighted the complex relationships between coping strategies, social support, psychological resilience, and mental health, thereby emphasizing the mediating roles of social support and psychological resilience in this context [87].

Although major hospitals have developed mental health strategies for nurses, research work and relevant policies seem to be limited with respect to the mental health of nurses working in primary care and nursing homes. Therefore, there is an urgent need to assess and address the impact of COVID-19 on nurses' mental health in nonhospital settings, as well as monitor international policies aimed at improving nurses' working conditions [40].

## 4. Conclusions

Several recent studies have clearly demonstrated that nurses, especially female nurses, caring for COVID-19 patients were the most vulnerable group of HCW, with related problems, including mental, psychological, and ethical issues that may have an impact on patient care. A positive attitude, job satisfaction, adequate relevant information, training, attention of their leaders, harmonious cooperation among team members, a sense of meaning in life, post-traumatic development, and emotional intelligence were factors that either improved or prevented psychopathology. In addition, psychological counseling, an increased access to personalized health care by psychotherapists and psychiatrists, psychiatric consultations through liaison meetings, stress management programs, sidewalk contact, and attention-based stress reduction could help reduce work-related problems.

Nurses must have easy access to such interventions and the stigma associated with psychiatric consultation must be removed. According to limited longitudinal studies, there is adaptability to the problems involved, with the level of pressure reduced at a later evaluation. Meanwhile, the changes in routine that followed were shown to enhance the adaptation process. In general, nurses require a strong moral support, as well as strong leadership from leaders who are adequately prepared for this responsibility. Additionally, they require clear information and directives, as well as strong support from anyone who can provide assistance.

## Use of AI tools declaration

The author declares no Artificial Intelligence (AI) tools have been used in the creation of this article.

## References

[1] Selye H (1950). Anxiety and the general adjustment syndrome. Br Med J.

[2] Chrousos GP (2009). Stress and stress system disorders. Nat Rev Endocrinol.

[3] Taylor S, Landry CA, Paluszek (2020). COVID stress syndrome: Concept, structure, and correlations. Depress Anxiety.

[4] Vizheh M, Qorbani M, Arzaghi SM (2020). The mental health of healthcare workers in the COVID-19 pandemic: A systematic review. J Diabetes Metab Disord.

[5] Veitch P, Richardson K (2021). Nurses need support during the Covid-19 pandemic. J Psychiatric Health Nurs.

[6] Stelnicki AM, Jamshidi L, Ricciardelli R (2021). Exposure to potentially psychologically traumatic events among nurses in Canada. Canadian J Nurs Res.

[7] Di Tella M, Romeo A, Benfante A (2020). Mental health of healthcare workers during the COVID-19 pandemic in Italy. J Eval Clin Pract.

[8] Lai J, Ma S, Wang Y (2020). Factors related to mental health outcomes among healthcare workers exposed to coronavirus disease 2019. JAMA Network Open.

[9] Nie A, Su X, Zhang S (2020). Psychological impact of the COVID-19 outbreak on frontline nurses: A cross-sectional research study. J Clin Nurs.

[10] Shahrour G, Dardas LA (2020). Acute stress disorder, coping with self-efficacy and subsequent psychological distress among nurses amid COVID-19. J Nurs Manag.

[11] Sarboozi Hoseinabadi T, Kakhki S, Teimori G (2020). Burnout and its influence factors among frontline nurses and nurses from other wards during the coronavirus disease epidemic -COVID-19- in Iran. Invest Educ Enferm.

[12] Liu Y, Long Y, Cheng, Y (2020). Psychological impact of the COVID-19 outbreak on nurses in China: A national survey during the epidemic. Front Psychiatry.

[13] Bouza E, Arango C, Moreno C (2023). Impact of the COVID-19 pandemic on the mental health of the general population and healthcare workers. Rev Esp Quimioter.

[14] Harris ML, McLeod A, Titler MG (2023). Health experiences of nurses during the COVID-19 pandemic: A mixed methods study. West J Nurs Res.

[15] Vázquez Sánchez MÁ, Ayllón Pérez V, Gutiérrez Sánchez D (2023). Professional grief among nurses in Spanish public health centers after caring for COVID-19 patients. J Jurs Scholarsh.

[16] Stubin CA (2023). Steps towards a resilient future nursing workforce. Int J Νurs Educ Scholarsh.

[17] Alimoradi Z, Jafari E, Lin CY (2023). Assessment of moral distress among nurses: A systematic review and meta-analysis. Nurs Ethics.

[18] Beck JG, Majeed R, Brown TA (2023). Understanding the role of COVID-19-related work-related stress and institutional betrayal in nurses' mental health: Some heroes wear scrubs. J Trauma Stress.

[19] Boone LD, Rodgers MM, Baur A (2023). A comprehensive review of factors and interventions that impact nurses' well-being and safety during a global pandemic. Evid Based Nurs Worldviews.

[20] Lee BEC, Ling M, Boyd L (2023). The prevalence of potential mental health disorders among hospital health workers during COVID-19: A systematic review and meta-analysis. J Affect Disord.

[21] Dos Santos Alves Maria G, de Oliveira Serpa AL, de Medeiros Chaves Ferreira C (2023). Impact of mental health on health professionals' sleep patterns during the COVID-19 pandemic in Brazil. J affect disord.

[22] Atay N, Sahin Bayindir G, Buzlu S (2023). The relationship between post-traumatic development and psychological resilience of nurses working in pandemic clinics. Int J Nurs Knowl.

[23] Adams TN, Ruggiero RM, North CS (2023). Addressing mental health needs among frontline healthcare workers during the COVID-19 pandemic. Chest.

[24] Phillips J, Alipio JK, Hoskins JL (2023). The experience of frontline nurses during the COVID-19 pandemic: A phenomenological study. West J Nurs Res.

[25] Chong YY, Frey E, Chien WT (2023). The role of psychological flexibility in the relationships between burnout, job satisfaction, and mental health among nurses in the fight against COVID-19: A two-area investigation. J Nurs Scholar.

[26] He C, Xing L, Lu Y (2023). Psychological distress and risk factors in frontline nurses dealing with COVID-19 in less severely affected areas. J Health Psychohospital Serv.

[27] Che H, Wu H, Qiao Y (2023). Association between long working hours and mental health among nurses in China under COVID-19 pandemic: Based on a large cross-sectional study. BMC Psychiatry.

[28] Sagherian K, Steege LM, Cobb SJ (2023). Insomnia, fatigue, and psychosocial well-being during the COVID-19 pandemic: A cross-sectional survey of hospital nursing staff in the United States. J Clin Nurs.

[29] Mao X, Dong W, Zhang J (2023). Mental health status and its related factors among female nurses in normalizing COVID-19 epidemic prevention and control in China. Front Public Health.

[30] Credland N, Griffin M, Hamilton P (2023). The impact of COVID-19 on mental health and wellness in critical care nurses-a longitudinal, qualitative study. Nurs Crit Care.

[31] Spruijt I, Cronin A, Udeorji F (2023). Respected but stigmatized: Healthcare workers caring for COVID-19 patients. PloS One.

[32] Vianna ECDC, Baptista RV, Gomes RS (2023). COVID-19 Pandemic: Analysis of health effects on emergency service nursing workers via a qualitative approach. Int J Environ Res Public Health.

[33] Ergin E, Ozbolat G (2023). Use of holistic, complementary and alternative methods of medicine by nurses against COVID-19 anxiety. Altern Ther Health Med.

[34] Baraka AAE, Ramadan FH, Hassan EA (2023). Predictors of stress, anxiety, and depression of critical care nurses in response to the COVID-19 pandemic. Nurs Care Rev.

[35] Almhdawi KA, Alrabbaie H, Arabiat A (2023). Quality of life and its health and occupational determinants among hospital-based nurses during the COVID-19 pandemic. Work.

[36] Yarifard K, Abravesh A, Sokhanvar M (2023). Work-family conflict, burnout, and related factors among nurses during the COVID-19 pandemic in northwestern Iran. Task.

[37] Kealeboga KM, Ntsayagae EI, Tsima O (2023). Psychological impact of COVID-19 on nurses caring for patients during the COVID-19 pandemic in Gaborone. Nurs Open.

[38] Khatatbeh H, Al-Dwaikat T, Alfatafta H (2023). Burnout, quality of life, and perceived patient adverse reactions among pediatric nurses during the COVID-19 pandemic. J Clin Nurs.

[39] Hwang S, Lee J (2023). The effect of COVID-19-related resilience on depression, work-related stress, sleep quality, and burnout among intensive care unit nurses. Psychol Front.

[40] García Vivar C, Rodríguez Matesanz I, San Martín-Rodríguez L (2023). Analysis of mental health impacts among nurses working during the COVID-19 pandemic: A systematic review. J Psychiatr Health Nurs.

[41] Sampaio F, Gaspar S, Fonseca C (2023). Sleep quality among nurses and the general population during the COVID-19 pandemic in Portugal: What are the differences?. Int J Environ Res Public Health.

[42] Chen R, Sun C, Chen JJ (2021). A large-scale survey on trauma, burnout, and post-traumatic development among nurses during the COVID-19 pandemic. Int J Mental Health.

[43] Sorokin MY, Kasyanov ED, Rukavishnikov GV (2020). Stress and stigma among healthcare workers during the COVID-19 pandemic. Indian J Psychiatry.

[44] Azizpour I, Mehri S, Moghaddam HR (2021). The impact of psychological factors on bereavement among frontline nurses fighting Covid-19. Int J Africa Nurs Sci.

[45] Costa A, Caldas de Almeida T, Fialho M (2023). Mental health of health professionals: Two years of COVID-19 pandemic in Portugal. Int J Environ Res Public Health.

[46] Padmanathan P, Lamb D, Scott H (2023). Suicidal thoughts and behaviour among healthcare workers in England during the COVID-19 pandemic: A longitudinal study. PloS One.

[47] González-Nuevo C, Postigo Á, González-Menéndez A (2023). Professional quality of life and COVID-19 fear among Spanish nurses: A longitudinal repeatable cross-sectional study. J Clin Nurs.

[48] Perego G, Cugnata F, Brombin C (2023). Analysis of the mental health of healthcare workers during the COVID-19 pandemic: Evidence from a three-wave longitudinal study. J Health Psychol.

[49] Heesakkers H, Zegers M, van Mol M M C (2023). Mental well-being of intensive care unit nurses after the second wave of the COVID-19 pandemic: A cross-sectional and longitudinal study. Intensive Crit Care Nurs.

[50] Pappa S, Ntella V, Giannakas T (2020). Prevalence of depression, anxiety, and insomnia among healthcare workers during the COVID-19 pandemic: A systematic review and meta-analysis. Brain Behav Immun.

[51] Ruiz Fernández MD, Ramos Pichardo JD, Ibáñez Masero O (2020). Compassion fatigue, exhaustion, compassion satisfaction, and perceived anxiety among healthcare professionals during the COVID-19 health crisis in Spain. J Clin Nurs.

[52] Badahdah A, Khamis F, Al Mahyijari N (2021). The mental health of healthcare workers in Oman during the COVID-19 pandemic. Int J Soc Psychiatry.

[53] Barello S, Palamenghi L, Graffigna G (2020). Burnout and physical symptoms among frontline health professionals at the height of the Italian COVID-19 pandemic. Psychiatry Res.

[54] Jo S, Kurt S, Bennett JA (2021). The resilience of nurses against coronavirus (COVID-19): An international view. Nurs Health Sci.

[55] Nguyen N, Le DD, Colebunders R (2021). Stress and related factors among frontline healthcare workers at the epicenter of COVID-19 in Da Nang, Vietnam. Int J Environ Res Public Health.

[56] Simonetti V, Della Pelle C, Cerratti F (2021). Presenteeism levels among Italian nurses. A multicentric survey. Prof Inferm.

[57] Hammond NE, Crowe L, Abbenbroek B (2021). Impact of the 2019 coronavirus disease pandemic on critical care healthcare workers' levels of depression, anxiety, and stress. Aust Crit Care.

[58] Bruyneel A, Gallani MC, Tack J (2021). Impact of COVID-19 on nursing time in intensive care units in Belgium. Intensive Crit Care Nurs.

[59] Kader N, Elhusein B, Al Abdulla S (2021). Risk perception and psychological impact of the COVID-19 pandemic among healthcare workers in primary and secondary healthcare settings in Qatar: A national study. J Prim Care Community Health.

[60] Bizri M, Kassir G, Tamim H (2021). Psychological distress experienced by doctors and nurses in a tertiary care center in Lebanon during the COVID-19 outbreak. J Health Psychol.

[61] Maraqa B, Nazzal Z, Zinc T (2021). Mixed method study to investigate ethical dilemmas and the willingness of healthcare workers to work amid the COVID-19 pandemic in Palestine. Front Med (Lausanne).

[62] Moore KS, Hemmer CR, Taylor JM (2021). Nurse stress level during coronavirus disease 2019: A looming workforce issue. J Pract Nurse.

[63] Brady C, Fenton C, Loughran O (2023). The mental health of Dublin hospital workers during the peak of Ireland's COVID-19 pandemic. Irish J Med Sci.

[64] Alfonsi V, Scarpelli S, Gorgoni M (2023). Healthcare workers after two years of COVID-19: The consequences of the pandemic on psychological health and sleep among nurses and doctors. Int J Environ Res Public Health.

[65] Tamrakar P, Pant SB, Acharya SP (2023). Anxiety and depression among nurses in COVID and non-COVID intensive care units. Nursing Care Review.

[66] Hegazy AA, Abdel Hamid TA, Zein MM (2023). Stress among healthcare providers in the NICU department, tertiary pediatric care hospital during the COVID-19 pandemic in Egypt. J Public health Res.

[67] Martin SD, Urban RW, Foglia DC (2023). Wellness in acute care nurse managers: Risk analysis of physical and mental health factors. Worldv Evid-based Nu.

[68] Canal Rivero M, Montes García C, Garrido Torres N (2023). The impact of the COVID-19 pandemic on the psychological well-being of healthcare workers: A 6-month longitudinal cohort study. Rev Psiquiatr Salud Ment.

[69] Kells M, Jennings Mathis K (2023). Impact of COVID-19 on the next generation of nurses in the United States. Jay Kline Nour.

[70] Sampaio F, Sequeira C, Teixeira L (2021). Impact of the COVID-19 outbreak on nurses' mental health: A prospective cohort study. Environment Res.

[71] Hendy A, Abozeid A, Sallam G (2021). Prognostic factors influencing stress among nurses providing care in COVID-19 isolation hospitals in Egypt. Nurs Open.

[72] Saifullah, Ma Z, Li M (2023). Impact of the COVID-19 pandemic on healthcare workers (HCW) in Sindh province, Pakistan. Health Res Policy Sy.

[73] Soto Rubio A, Giménez Espert MDC, Prado Gascó V (2020). Impact of emotional intelligence and psychosocial risks on nurses' burnout, job satisfaction, and health during the COVID-19 pandemic. Int J Environ Res Public Health.

[74] Franco J A, Leví P L Á (2020). Feelings, Stress, and Adaptation Strategies of Nurses against COVID-19 in Guayaquil. Invest Educ Enferm.

[75] Nowicki GJ, Ślusarska B, Tucholska K (2020). The severity of traumatic stress associated with the COVID-19 pandemic, perception of support, sense of security, and sense of meaning in life among nurses: Research protocol and preliminary results from Poland. Int J Environ Res Public Health.

[76] Letvak S, Ruhm C, Lane S (2011). The impact of nurses' health on productivity and quality of care. J Nurs Adm.

[77] Janeway D (2020). The Role of Psychiatry in Treating Burnout Among Nurses During the Covid-19 Pandemic. J Radiol Nurs.

[78] Labrague LJ, De Los Santos JAA (2021). Prevalence and prognostic factors of coronaphobia among frontline hospital and public health nurses. Public Health Nurses.

[79] Kim MY, Yang YY (2021). Mental health status and factors affecting it: the case of nurses working in COVID-19 hospitals in South Korea. Int J Environ Res Public Health.

[80] Al Dossary R, Alamri M, Albaqawi H (2020). Awareness, attitudes, prevention, and perceptions of the COVID-19 epidemic among nurses in Saudi Arabia. Int J Environ Res Public Health.

[81] Turale S, Meechamnan C, Kunaviktikul W (2020). Hard times: Ethics, nursing, and the COVID-19 pandemic. Int Nurs Rev.

[82] Hofmeyer A, Taylor R (2021). Strategies and resources that nurse leaders can use to lead with empathy and prudence to understand and address the sources of stress among practicing nurses in the COVID-19 era. J Clin Nurs.

[83] Dimino K, Learmonth AE, Fajardo CC (2021). Nursing managers lead the way: they reimagine stress to maintain a healthy work environment. Nurse Crit Care.

[84] White JH (2021). A phenomenological study of the experiences of nursing managers and assistant nursing managers during the COVID-19 pandemic in the United States. J Nurs Manag.

[85] Lin CH, Siao SF, Lin YJ (2023). Cognitive assessments and coping strategies of enrolled nurses in the emergency department to combat COVID-19: A delineation review. J Nurs Scholarsh.

[86] Lim S, Park H, Kim S (2023). Psychological experiences of nurses caring for COVID-19 patients: A comprehensive review based on qualitative research. Nurs Open.

[87] Xu Y, Zheng QX, Jiang XM (2023). Effects of coping on nurses' mental health during the COVID-19 pandemic: Mediating role of social support and psychological resilience. Nurs Open.

